# Utilization of the philtral pressure technique as an efficacious measure to prevent coronavirus transmission through sneezing

**DOI:** 10.5935/0004-2749.20200112

**Published:** 2024-02-11

**Authors:** Eugene R. Ting, Elizabeth L.S. Wong, Michael Lin, Tyler Blah, Jessica Huang, Pragya Goswami, Yi Yang, Muhammad A. Khan, Zhi Wei Lim, Ashish Agar, Ian C. Francis

**Affiliations:** 1 University of New South Wales, Sydney, Australia; 2 Department of Ophthalmology, Prince of Wales Hospital, Sydney. Australia; 3 University of Notre Dame, Sydney, Australia; 4 Royal North Shore Hospital, Sydney, Australia

The initial cases of the novel coronavirus (COVID-19) were first reported from Wuhan, China, in late December 2019. The spread of COVID-19 has since been exponential. The World Health Organization (WHO) reports that the reproductive number (R0) of COVID-19 is significant, with an estimated R0 of 1.4-2.5, similar to that of the Spanish flu of 1918^1^. As of the third week of August 2020, 23.1 million cases and more than 803,000 deaths have occurred worldwide, with more than 113,000 deaths in Brazil alone^(1)^.

As human-to-human spread through respiratory droplets and direct contact are the primary route of transmission of this potentially fatal illness, guidelines for mitigation of the COVID-19 spread by the Centers for Disease Control and the WHO have been proposed. These include social distancing, wearing of facial masks, hand hygiene, sneezing into the inside of one’s elbow^(1^,^2)^, and covering one’s nose and mouth with a bent elbow or paper tissue when coughing or sneezing.

Despite these recommendations, the data pertaining to virus shedding from respiratory droplets and non-pharmaceutical interventions are limited. Guidelines for respiratory hygiene and sneeze etiquette have been made on the basis of plausible effectiveness rather than on controlled studies^(2)^.

The current literature shows that a single sneeze produces thousands of respiratory droplets capable of transmitting the virus. As the result of a sneeze, aerosolized particles may travel a long distance, around three times more than that which occurs with a cough^(3)^. A sneeze with an outlet velocity of 20 m/s can transport respiratory particles up to 3 m^(3)^. These particles do not have to be inspired to transmit COVID-19, and mucosal contact involving the eyes, mouth, or nose may result in infection.

Sneezing is a physiologically coordinated respiratory reflex in response to irritation in the nasal cavity. It results when afferent nerve fiber signals are transmitted via the ethmoidal, ophthalmic, and maxillary branches of the trigeminal nerve to the trigeminal nerve nuclei in the pons. The efferent nerve fiber signals travel to different parts of the body, resulting in deep inspiration and forced expiration with initial closing of the glottis^(4)^. This results in increased intrapulmonary pressure, precipitating into a sneeze.

The photic sneeze reflex (PSR) is described as an uncontrollable paroxysm of sneezing provoked by the sudden exposure of a dark-adapted person to an external light stimulus^(4)^. The PSR is in fact a complex neuro-ophthalmological phenomenon, involving the optic, ocular motor, and trigeminal nerves, as well as autonomic pathways and the central brainstem. It is postulated that the PSR occurs as a result of optic-trigeminal summation. Here persistent light exposure relays signals via the optic and trigeminal nerves, which leads to increased sensitivity in the maxillary branch of the trigeminal nerve, precipitating the urge to sneeze. An alternative mechanism for the PSR has been described by Everett et al.^(5)^ through parasympathetic generalization, where light exposure not only results in ocular sensory input but also causes the activation of the neighboring neurons involved in the sneeze reflex.

**Figure 1:**
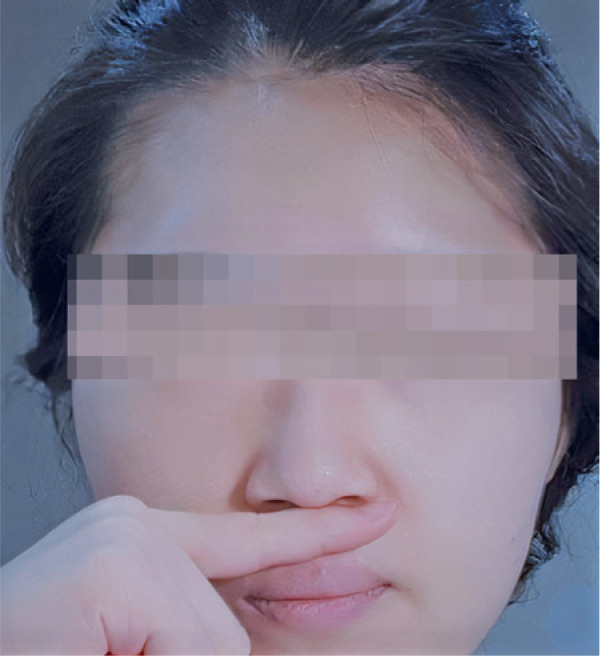
Philtral pressure technique: For the purposes of the photograph, the subject applies a firm digital pressure transversely to the skin of the subphiltral region, directed posterosuperiorly onto the maxilla, inferior to and abutting the inferior nasal spine.

The philtral pressure technique (PPT), a simple technique previously described by the authors in the management of PSR, reliably prevents the urge to sneeze^(4)^. It involves the application of a firm digital pressure transversely to the skin of the subphiltral region, directed posterosuperiorly onto the maxilla, inferior to and abutting the inferior nasal spine (Figure 1). This stimulates the local mechanoreceptors, overriding the trigeminal nerve irritation. Alternatively, it may interfere with the coactivation of neighboring parasympathetic fibers. Whatever the case, the PPT almost always prevents sneezing^(4)^.

**Figure 2:**
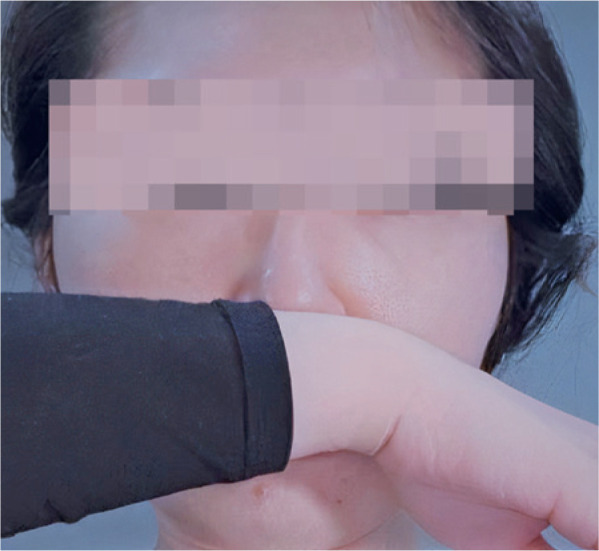
Modified philtral pressure technique. The subject uses the wrist to apply philtral pressure to prevent an imminent sneeze.

In the management of the COVID-19 pandemic, sneezing into one’s elbow has been recommended. Our group considers that in the first instance, attempting to prevent a sneeze is a superior approach. Furthermore, the PPT can be modified by using the wrist or distal forearm to apply philtral pressure (Figure 2), as this prevents the individual from touching the face with the hands.

Controlled studies to evaluate the effectiveness, cost, and transmissibility of COVID-19 and other non-pharmacological measures are necessary but have not yet been conducted. Along with the other recommended social and hygiene measures, utilization of the PPT may also assist in reducing the spread of COVID-19. Our group considers that if this straightforward technique is implemented rapidly in pandemic regions, substantial benefit could be derived not only for individuals but also for nations.
